# Classic Dissection of Thoracic Aorta Complicated by Ascending Aortic Intramural Hematoma: Promt Diagnosis and Successful Endovascular Repair

**DOI:** 10.1155/2012/257893

**Published:** 2012-01-12

**Authors:** Gediminas Rackauskas, Mindaugas Mataciunas, Nerijus Misonis, Diana Zakarkaite, Marijus Gutauskas, Valdas Bilkis, Algirdas Edvardas Tamosiunas, Pranas Serpytis, Aleksandras Laucevicius

**Affiliations:** ^1^Department of Cardiovascular Medicine, Vilnius University Hospital Santariskiu Klinikos, 08661 Vilnius, Lithuania; ^2^Department of Tomography, Vilnius University Hospital Santariskiu Klinikos, 08661 Vilnius, Lithuania; ^3^Department of Vascular Surgery, Vilnius University Hospital Santariskiu Klinikos, 08661 Vilnius, Lithuania

## Abstract

We reported a case of 68-year-old man, with a previous history of hypertension. Patient was admitted to our institution for evaluation of a severe, constant, tearing anterior chest pain radiated to the neck with suspicion of acute aortic dissection. A multidetector computed tomography scan of thorax and abdomen demonstrated a dissection starting from the middle part of aortic arch and extending downward to the descending aorta till the middle part of the thoracic aorta. The dissection was classified as Stanford A, De Bakey I. Surgical treatment of patient was started with bypass graft from the right common carotid artery to the left common carotid with subsequent revascularization of left subclavian artery. Lower parts of above-mentioned arteries were ligated. At the second stage an emergent prosthetic stent-graft was placed distally from the truncus brachiocephalicus up to the proximal part of the descending aorta. We reported a case report to present diagnostic and possible interventional treatment for patient with acute aortic type A dissection.

## 1. Introduction

Acute aortic dissection (AAD) is a medical emergency with a high mortality rate, rising by approximately 1% per hour. It usually presents with severe, unrelenting chest pain of sudden onset. Pain can be described as ripping or tearing in nature or stabbing or sharp in character. It can migrate as the dissection extends down to the aorta (17%). Less common signs and symptoms are related to organ hypoperfusion and include peripheral ischemic syndromes (19%), syncope (13%), myocardial infarction (13%), heart failure (8.8%), and neurologic symptoms (6.1%) [1–3]. Cases of painless AAD have also been described [4, 5]. Because of such heterogeneous clinical presentations, many cases still remain incorrectly diagnosed. Approximately one-third of patients ultimately diagnosed with AAD have another initial diagnosis. The true incidence of AAD is therefore difficult to determine, but is estimated to be 5–20/1,000,000 [6]. AAD is classified according to the location and extent of dissection. The two most commonly used classification systems are the DeBakey system, which categorizes aortic dissection by the location of original intimal tear and the extention of the dissection (type I–III), and the functional Stanford system, categorizing dissections into those involving the ascending aorta (type A, more common) and those distal to the outlet of the left subclavian artery (type B). In general, type A dissections have a higher in-hospital mortality rate (30%) and require immediate surgical treatment, whereas type B dissections have a better prognosis (10% in-hospital mortality) and may be managed conservatively. The clinical outcome of AAD is eventually determined by dissection type and timing of presentation, patient-related factors, and the quality and experience of the individuals and institution providing care [1]. 

## 2. Case Report

A 68-year-old male, with a history of hypertension, was emergently admitted to our university hospital because of strong, pressing, tearing chest pain (pain intensity 7 points from 10 according to visual-analogue scale), radiating to the neck, with a suspicion of acute aortic dissection.

**Figure 1:**
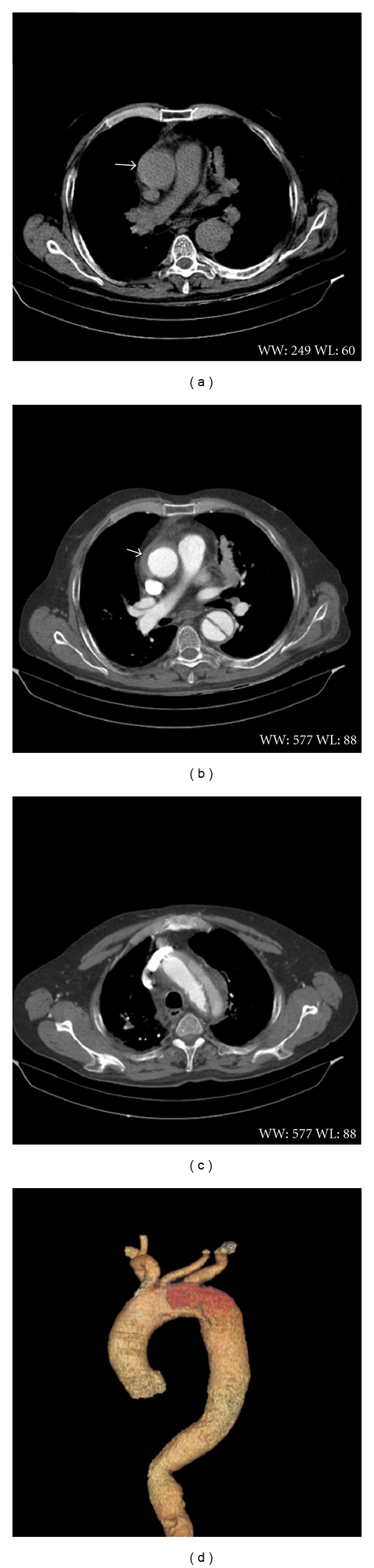
((a) and (b)) Preoperative computed tomography images demonstrating intramural hematoma in the ascending part of aorta. (c) Reveal classic dissection of the aortic arch with false and true lumen. (d) Reconstructed view.

Physical examination in the intensive care unit showed; stable haemodynamics blood pressure 154/76 mmHg; pulse was 87 beats per minute. No pulse deficit of radial artery was observed. Normal constitutional type—body mass index (BMI): 24 kg/m^2^. An electrocardiogram showed left ventricular hypertrophy. Laboratory workup revealed increased C reactive protein 72.4 mg/L (normal value <5 mg/L) and D-dimers 6825 mcg/L (normal value <250 mcg/L). Chest X-ray showed widened mediastinum, aneurism of aorta with abnormal contour. Heart ultrasound demonstrated hypertrophy of left ventricle and septum, EF-55%, second degree insufficiency of aorta valve. Small amount of fluid was observed in the pericardium. Ascending aorta was 47 mm diameter and had double contour with ~6 mm thickness anechogenic space. A thoracic and abdominal CT angiography demonstrated classic dissection of the aortic arch with false and true lumen starting distally to the brachiocephalic trunk and extending downward to the middle part of the thoracic descending aorta (Figures 1(b) and 1(c)). Location of the entry tear near the common left carotid artery was confirmed. True lumen of the distal aortic arch gave rise to the left common carotid artery and left subclavian artery (Figure 1(d)). Circular, crescent thickening of the ascending aortic wall on CT angiographic images well corresponded with the subtle circular rim of increased density (up to 60 HU) in nonenhanced images suggesting life-threatening acute aortic syndrome—intramural hematoma (Figures 1(a) and 1(b)).

The patient was treated to reduce blood pressure and was felt at risk for impending aortic rupture. Reduction of blood pressure with antihypertensive medication was started immediately; nevertheless, the risk of impending aortic rupture was evident. Therefore, a hybrid approach with endovascular stenting was considered. In this case surgical strategy included right common carotid to left common carotid bypass and bypass graft from the right common carotid artery to the left common carotid with subsequent revascularization of left subclavian artery before placement of endovascular stent-graft on the aortic arch and descending aorta.

Under general anesthesia bilateral common carotid arteries and left subclavian arteries were explored through bilateral supraclavicular incision. Bypass was performed with (Twillweave Woven Vascular prosthesis, Renfrewshire, Scotland 8 mm × 100 mm) graft. The proximal parts of left common carotid and left subclavian arteries were ligated to avoid an endoleak over time after endovascular stent deployment. After this surgical intervention patients' haemodynamic state became unstable—drop of blood pressure was observed and sympatomimetics—and artificial lung ventilation was started for the next 36 hours and thereafter discontinued.

At 24 hours after operation focal neurological symptoms appeared, including positive Babinski's sign, however, brain-computed tomography showed no acute or chronic pathological lesions. These neurological changes were interpreted as transient ischemic disturbances in the left middle cerebral artery.

**Figure 2:**
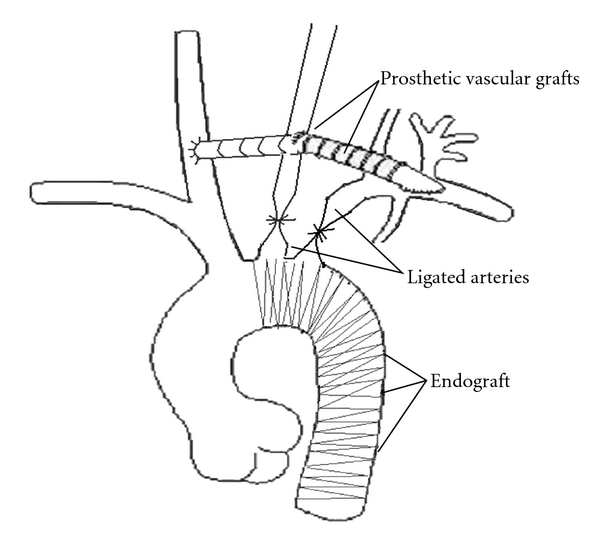
Illustration of the surgical technique. The carotid-to-carotid bypass with subsequent revascularization of left subclavian artery with the proximal segments ligation of the left carotid and left subclavian arteries. Endovascular stenting on the aortic arch and aortic debranching.

At the second stage of hybrid operation, aortic arch debranching with stent-graft placement was performed after preservation of cerebral circulation with the placement of graft from right common carotid to left common carotid artery. The endovascular stent-graft was introduced retrogradely through the right femoral artery. The stent-graft (Valiant, Medtronic, Sunnyvale, CA USA 44 mm × 150 mm) was deployed when it was in the proper position under angiogram. Our goal was to exclude the damaged segment of the aorta and to obliterate the entry of blood into the false lumen (both at the initial intimal tear and at the secondary tears along the vessel) (Figure 2). Postoperative course was uneventful; patient underwent rehabilitation and was discharged home after 14 days with antihypertensive treatment.

**Figure 3:**
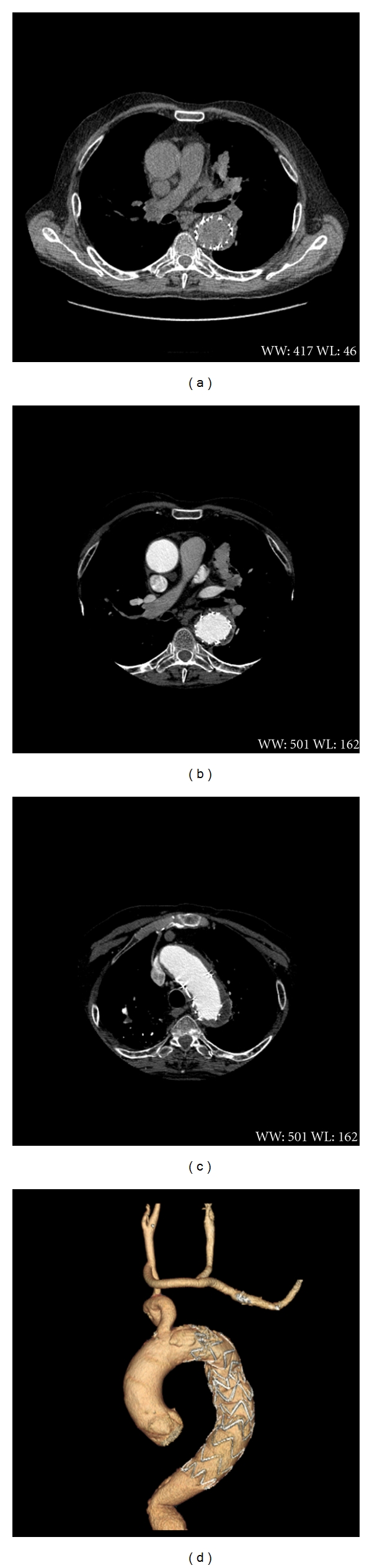
Computed tomographic angiogram at postoperative four weeks later. ((a) and (b)) No observation of intramural hematoma. (c) No observation of true and false lumen. (d) Reconstructed view of the aorta and endograft.

Four weeks later, blood pressure was controlled (130/78 mmHg) with antihypertensive therapy. Follow-up CT scan demonstrated the correct graft positioning, closure of the entry tear and homogenous enhancement of the branches of the aortic arch (Figures 3(b), 3(c), and 3(d)). Nonenhanced images showed no signs of intramural hematoma.

## 3. Discussion

When the diagnosis of acute aortic dissection is established, it is necessary to control pain, reduce systolic blood pressure to values between 100 and 120 mmHg, heart rate to decrease the shear forces on the dissected aorta. Intravenous beta-blockers (e.g., labetolol or metoprolol) are the mainstay of medical treatment. If needed, vasodilatation with agents such as sodium nitroprusside or intravenous calcium channel blockers can be used. Patients with profound hemodynamic instability need to be intubated and ventilated without delay.

 The aims of surgical intervention in type A acute aortic dissection are to prevent aortic rupture and pericardial effusion leading to cardiac tamponade, to relieve aortic regurgitation, and to prevent myocardial ischemia. A large variability of surgical techniques exists, depending on the anatomic conditions and the condition of the aortic valve [7].

 Intramural hematoma of the ascending aorta has a prognosis similar to type A dissection. Progression to classic aortic dissection occurs in 28% to 47% of patients and may carry a risk of rupture in 20% to 45% of cases [8]. There is evidence that extention of intramural hematoma depends on specific location of culprit plaque in the wall of thoracic aorta [9]. If in the convexity of the distal arch, supra-aortic branches prevent retrograde extension toward the ascending aorta. If at the free lateral wall or at the concavity, intramural hematoma may affect the entire thoracic aorta, owing to the lack of the natural barrier of the supra-aortic branches. Endovascular stent-graft placement of this plaque-associated intramural hematoma may be more effective and less invasive than conventional surgery to treat the entire thoracic aortic disease [9]. In our patient culprit plaque was not identified; however, dissection extending up to the left common carotid artery and intramural hematoma of ascending aorta suggested high-risk variant with worse prognosis directing our decision on management strategy.

Surgical intervention is indicated in all patients with proximal dissections, with the exception of patients with serious concomitant conditions that preclude surgery. Stroke is often a contraindication for surgery because there is real concern that anticoagulation therapy and reperfusion can result in further neurological deterioration by converting the ischemic stroke to a hemorrhagic stroke [10]. The perioperative mortality rate for patients with aortic dissections ranges from 5 to 10% and may approach 70% in cases with complications [11].

 The carotid-to-carotid bypass is technically much simpler and more expeditious than the intrathoracic supra-aortic bypass procedures through sternotomy. The main advantage is the avoidance of potential complications and aortic clamping [12]. The avoidance of aortic clamping prevents systemic embolization and associated complications, such as cerebral ischemia and traumatic aortic injury. This method can be beneficial in patients with advanced physiologic age, multiorgan dysfunction, prior operative procedures or incision site infections that make surgical exposure difficult, diabetes, and chronic obstructive lung disease.

In the literature, the subclavian-to-subclavian bypass was also noted for extra-anatomic hybrid endovascular repair of the aortic arch pathologies. The subclavian-to-subclavian bypass before stent-graft placement decreases the risk of ischemia of the upper extremity and allows preservation of the left internal thoracic artery for a coronary surgery in the future. Potential disadvantages of the subclavian-to-subclavian bypass may be related to cosmetic problems: its pulsation can be visible and palpable above manubrium. The graft can be subject to an injury in the pretracheal position and disturbs the exploration for a tracheostomy in the future. This type of bypass is contraindicated in patients with brachiocephalic artery lesions [13].

 Clinical outcome is determined by a variety of factors; the key in management of acute aortic dissection is to maintain a high level of suspicion for this diagnosis.
